# ω-3 Polyunsaturated Fatty Acids Improve the Blood–Brain-Barrier Integrity in Contrast-Induced Blood–Brain-Barrier Injury in Uremic Mice

**DOI:** 10.3390/ijms241512168

**Published:** 2023-07-29

**Authors:** Jin Ah Shin, Hyerim Park, Hyunsu Choi, Yoon-Kyung Chang, Jwa-Jin Kim, Young Rok Ham, Ki Ryang Na, Kang Wook Lee, Dae Eun Choi

**Affiliations:** 1 Department of Medical Science, Chungnam National University, Daejeon 35015, Republic of Korea; wlsdkahh@gmail.com (J.A.S.); hye05240@gmail.com (H.P.); 2 Clinical Research Institute, Daejeon Saint Mary Hospital, Daejeon 34943, Republic of Korea; peace420@cmcdj.or.kr; 3 Department of Nephrology, Daejeon Saint Mary Hospital, Daejeon 34943, Republic of Korea; racer@catholic.ac.kr; 4 Department of Nephrology, Chungnam National University Hospital, Daejeon 35015, Republic of Korea; kjj4827@gmail.com (J.-J.K.); drngr@cnu.ac.kr (K.R.N.); kwlee@cnu.ac.kr (K.W.L.)

**Keywords:** ω-3 PUFA, ischemia–reperfusion, blood–brain barrier (BBB), uremic toxin, contrast media (CM)

## Abstract

In patients with chronic kidney disease, the need for examinations using contrast media (CM) increases because of underlying diseases. Although contrast agents can affect brain cells, the blood–brain barrier (BBB) protects against brain-cell damage in vivo. However, uremia can disrupt the BBB, increasing the possibility of contrast-agent-induced brain-cell damage in patients with chronic kidney disease (CKD). ω-3 polyunsaturated fatty acids (PUFAs) have shown protective effects on various neurological disorders, including uremic brain injury. This study examined whether ω-3 PUFAs attenuate damage to the BBB caused by uremia and contrast agents in a uremic mouse model and evaluated its associated mechanisms. C57BL/6 mice (eight weeks old, male) and fat-1 mice (b6 background/eight weeks old, male) were divided into groups according to uremic induction, CM, and ω-3 PUFA administration. Uremia was induced via 24 h ischemia–reperfusion (IR) renal injury. One day after CM treatment, the brain tissue, kidney tissue, and blood were collected. The expression levels of glial fibrillary acidic protein (GFAP), claudin 5, CD31, laminin α4, and laminin α5 increased in ω-3 PUFA + CM-treated uremic mice and the brain of fat-1 + CM-treated uremic mice compared with those in the brains of CM-treated uremic mice. The pro-apoptotic protein expression decreased, whereas the anti-apoptotic proteins increased in ω-3 PUFA + CM-treated uremic mice and fat-1 + CM-treated uremic mice compared with CM-treated uremic mice. In addition, the brain-expression levels of p-JNK, p-P53, and p-P38 decreased in the ω-3 PUFA + CM-treated uremic mice and fat-1 + CM-treated uremic mice compared with those in wild-type uremic mice. Our results confirm that uremic toxin and CM damage the BBB and cause brain-cell death. ω-3 PUFAs play a role in BBB protection caused by CM in uremic mice.

## 1. Introduction

Complications of intravascular administration of contrast media (CM) include anaphylactic reactions, cardiac arrhythmias, acute renal failure, and neurotoxic effects. The well-known side effects of CM are localized in the kidneys through multiple mechanisms; however, the incidence of severe neurotoxicity is low [1,2]. CM directly increases cytotoxicity in the proximal tubule of the kidney, causing renal damage such as increased cell damage through reactive oxygen species and increased blood-flow resistance in the kidney [1,3]. CM causes neurotoxicity. However, its incidence is low in the normal brain parenchyma because the blood–brain barrier (BBB) is impervious to large molecules such as intact and iodized contrast agents [4].

At the level of the cerebral microvascular endothelium, the BBB, a selective diffusion barrier, is not significantly affected by CM administration, whereas renal failure and associated uremia affect the cardiovascular system, brains, and BBB [5]. In patients with chronic kidney disease (CKD), the lack of renal clearance results in the accumulation of uremic solutes in the circulation, some of which are considered uremic toxins and believed to contribute to uremic syndrome [4,6]. The disruption of endothelial tight-junction proteins can lead to BBB damage in patients with uremia, and exposure and retention of uremic toxins, altered metabolism, and inflammatory and vascular changes are believed to result in BBB dysfunction [7,8]. Taken together, these results indicate that CKD may cause BBB fragmentation due to CM. As such, the induction of CKD leads to BBB dysfunction; however, the mechanism for BBB destruction is not fully explained when CM is administered in patients with CKD.

Eicosapentaenoic acid (EPA) and docosahexaenoic acid (DHA), the two main biologically active ingredients of ω-3 polyunsaturated fatty acids (PUFAs), prevent numerous metabolic disorders. ω-3 PUFAs benefit various neurological disorders because of their anti-inflammatory, antioxidant, apoptosis-prevention, and neuroprotective effects [9,10]. The destruction of the BBB plays an important role in cell damage in neurological diseases, including acute and chronic brain ischemia, brain trauma, multiple sclerosis, brain tumors, and brain infections. Researchers have described the pathogenic mechanisms leading to nerve damage, synaptic dysfunction, loss of neural connectivity, and neurodegeneration [11,12]. This showed that BBB damage has long been involved in neurodegeneration and that ω-3 PUFA-rich diets reduce BBB destruction and brain nerve loss [13].

Jun N-terminal kinase (JNK) and p38 mitogen-activated protein kinase (MAPK) play a central role in oxidative stress-induced apoptosis and contribute to neuroinflammation, cerebrovascular system, and BBB removal [14,15,16,17]. DHA, a major component of ω-3 PUFAs, inhibits JNK/p38 MAPK phosphorylation by reducing the production of infectious cytokines such as tumor necrosis factor-α, interleukin (IL)-1β, and IL-6 in neuroglia cells [18,19]. Inhibited increase in reactive-oxygen-species [12] accumulation with EPA treatment, another major component, was found to be accompanied by the inhibited activation of p38 and JNK kinases and attenuation of subsequent apoptosis [20]. These findings suggest neural protection through reduced activation of p38 and JNK kinase alone with EPA and DHA in endothelial cells [21].

Given the above background, this study aimed to investigate novel molecular factors in CKD that contribute to BBB damage, determine whether ω-3 PUFAs ameliorate CKD-induced brain damage, and evaluate the molecular mechanisms involved.

## 2. Results

### 2.1. Ischemia–Reperfusion-Induced Renal Damage Induces Uremic Production

**Figure 1:**
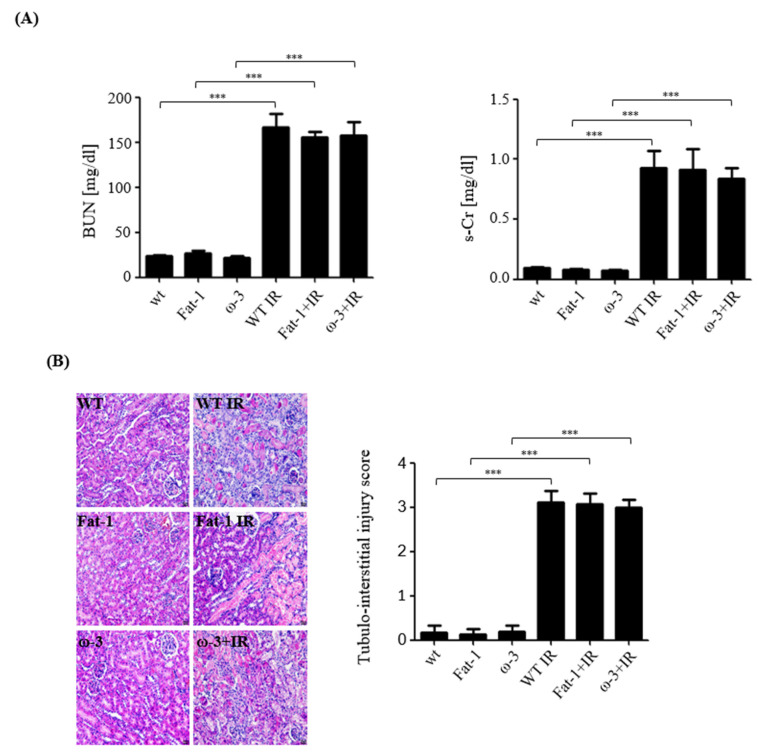
Renal function and histology after ischemic–reperfusion (IR) renal injury. Renal function (WT, wild-type sham; fat-1, fat-1 induction sham; ω-3, ω-3 PUFA oral-administration sham; IR, IR renal injury in wild-type mice; fat-1 + IR, IR renal injury in fat-1 induction mice; ω-3 + IR, IR renal injury in ω-3 PUFA orally administered mice.) (**A**) The levels of blood urea nitrogen (BUN) and serum creatinine (s-Cr) were significantly increased in the IR group compared with those in each control group. (**B**) Representative kidney section with hematoxylin and eosin staining. Tubulointerstitial injuries represent dilated renal tubules and tubular necrosis and inflammatory cells. Original magnification, 200×. Scale bar = 50 μm. The bar represents mean ± SD. *** *p* < 0.001.

Blood urea nitrogen (BUN) and serum creatinine were measured to evaluate kidney function. The levels of BUN and s-Cr after IR renal injury was significantly increased compared with those of the control group (Figure 1A). Hematoxylin and eosin (H&E) staining showed tubular cell necrosis and detachment and interstitial inflammatory cell infiltration in IR mice kidney. The tubulointerstitial injury score was significantly increased in IR mouse kidney, compared with each control (Figure 1B).

### 2.2. Effects of CM Administration on Hippocampal Nerve Damage after Renal IR

**Figure 2:**
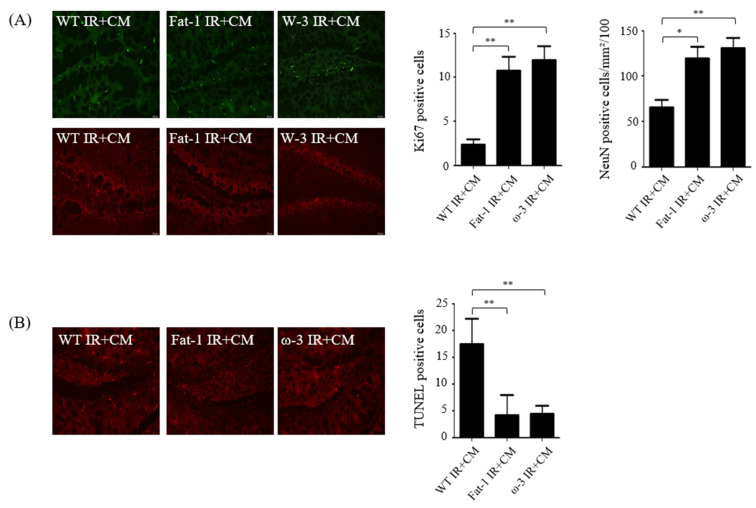
Hippocampal damage in ischemia–reperfusion (IR) kidney. (**A**) Representative immunofluorescence-stained brain section. Immunofluorescent staining was performed using the cell proliferation marker Ki67 and neuronal nuclei marker (NeuN) in the brain. Ki67 and NeuN expression significantly increased in the brains of WT IR + CM mice. Original magnification, 200×. Scale bar = 50 μm. (**B**) Representative TUNEL-stained brain section. The number of TUNEL-positive cells increased in the brains of WT IR + CM mice. Original magnification, 200×. Scale bar = 50 μm. Bar represents mean ± standard deviation. IR + CM, IR renal injury in wild-type mice administered with CM; fat-1 IR + CM, IR renal injury in fat-1 induction mice administered with CM; ω-3 IR + CM, IR renal injury in ω-3 PUFA orally treated mice administered with CM. * *p* < 0.05, ** *p* < 0.01.

To determine whether damage occurred in the brain hippocampus of uremia mice, immunofluorescence staining was performed using proliferating cell marker Ki67 and neuronal nuclei marker NeuN. Compared with the brains of fat-1 IR + CM and ω-3 IR + CM uremic mice, the expression level of Ki-67 was reduced in the brains of wild-type (WT) IR + CM uremic mice. The differentiation and developmental intensity of the cranial neural network were also lower in the brains of IR + CM than in those of fat-1 IR + CM and ω-3 IR + CM uremic mice (Figure 2A). Neuronal terminal deoxynucleotidyl transferase dUTP nick end labeling (TUNEL) analysis was performed to evaluate whether cell death occurred in the brains of uremic mice. The number of neuron TUNEL-positive cells was higher in the brains of uremic mice than in those of fat-1 IR + CM and ω-3 IR + CM uremic mice (Figure 2B).

### 2.3. In Non-Uremic Mouse Brain, CM Administration Has No Effect

To verify whether CM administration alone affects brain damage, WT, fat-1 WT, and ω-3 PUFA WT were compared with WT + CM, fat-1 WT + CM, and ω-3 PUFA WT + CM. Immunofluorescence staining was performed using the astrocyte marker GFAP, pericyte marker CD31, endothelial cell marker claudin 5, and extracellular matrix (ECM) markers laminins α4 and α5. No significant difference was found in the expression levels of markers in the brains of CM-treated WT, fat-1 WT, and ω-3 PUFA WT mice. No difference was found in the number of TUNEL-positive cells in the brains of WT, fat-1 WT, and ω-3 PUFA WT mice and the brains of WT + CM, fat-1 WT + CM, and ω-3 PUFA WT + CM mice (not shown).

### 2.4. CM-Induced BBB Injuries Are Attenuated in Fat-1 Uremic Mice

**Figure 3:**
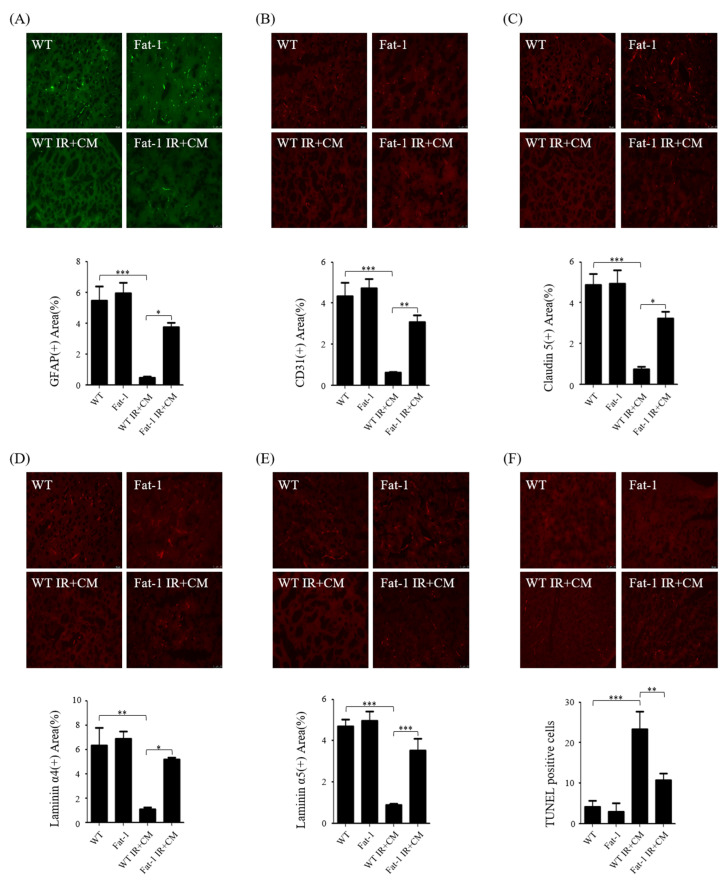

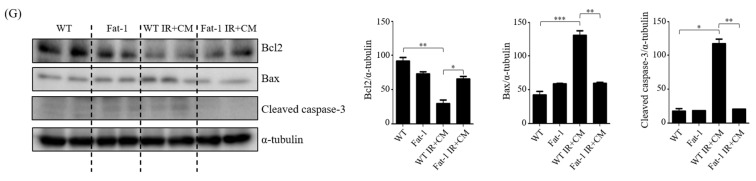
ω-3 PUFAs protect against CM in the brains of IR-injured fat-1 mice. (**A**–**E**) Representative immunofluorescence-stained brain section. Immunofluorescence staining was performed in the brains using the astrocyte marker GFAP, pericyte marker CD31, endothelial cell marker claudin 5, and extracellular matrix (ECM) markers laminin α4 and α5. Original magnification, 200×. Scale bar = 50 μm. (**F**) Representative TUNEL-stained brain section. Original magnification, 200×. Scale bar = 50 μm. (**G**) Representative Western blot of brain lysis: brain of fat-1 IR + CM shows decreased Bax, cleaved caspase-3, and increased Bcl2 expression compared with the brain of WT IR + CM. WT IR + CM. Bar represents mean ± SD. WT, wild-type sham; fat-1, fat-1 induction sham; WT IR + CM, IR renal injury in wild-type mice administered with CM; fat-1 IR + CM, IR renal injury in fat-1 induction mice administered with CM. * *p* < 0.05, ** *p* < 0.01, *** *p* < 0.001.

We evaluated CM-induced BBB injuries in uremic fat-1 mice. First, immunofluorescence was performed with astrocyte marker GFAP, pericyte marker CD31, endothelial cells marker claudin 5, and ECM markers laminin α4 and α5. Compared with the brains of WT and fat-1 WT mice, the expression levels of the markers were decreased in the brains of each group of WT IR + CM mice but increased in the brains of fat-1 IR + CM compared with WT IR + CM mice (Figure 3A–E). The number of TUNEL-positive cells was increased in the brains of WT IR + CM and fat-1 IR + CM mice compared with that in the brains of WT and fat-1 WT mice; however, the number of TUNEL-positive cells decreased in the brains of fat-1 IR + CM mice compared with that in the brains of WT IR + CM mice (Figure 3F). In addition, the protein expression of Bax and cleaved caspase-3 were significantly decreased in the brains of fat-1 IR + CM mice compared with the expression in the brains of WT IR + CM mice. However, Bcl2 expression significantly increased in the brains of fat-1 IR + CM mice compared with that in the brains of WT IR + CM mice (Figure 3G).

### 2.5. ω-3 PUFAs Attenuate BBB Injury via Contrast in Uremic Mice

**Figure 4:**
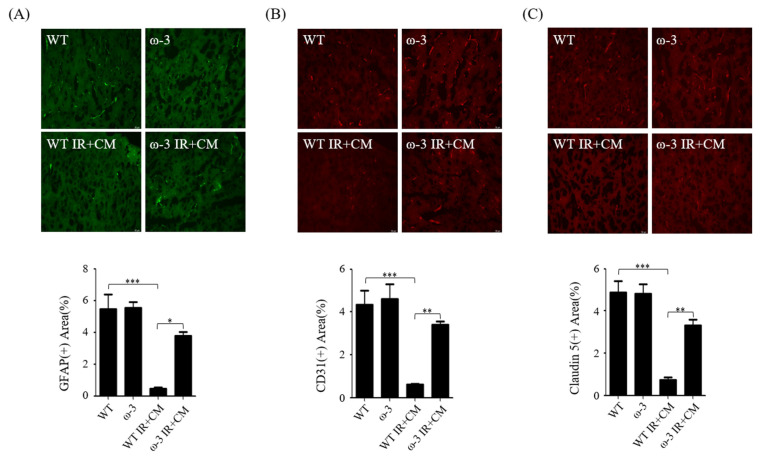

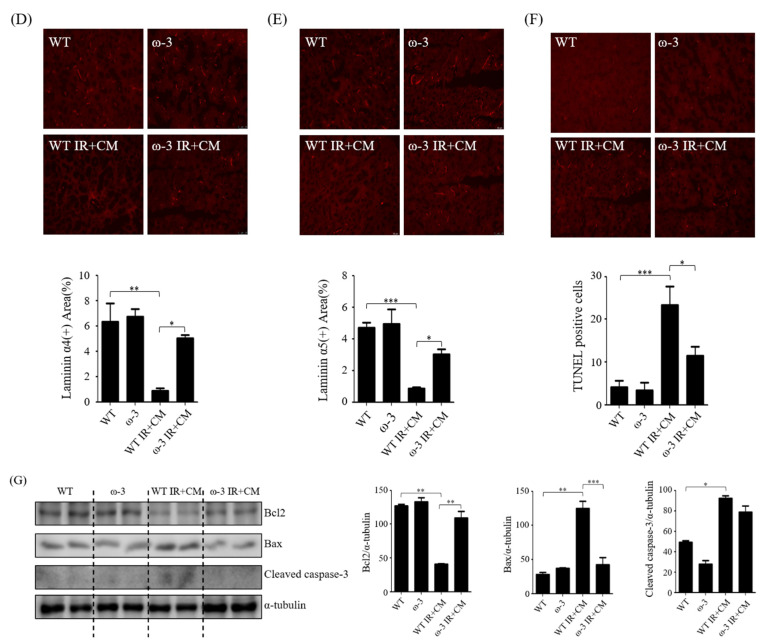
ω-3 PUFAs protect against CM in the brains of IR-injured ω-3 PUFA mice. (**A**–**E**) Representative immunofluorescence-stained brain section: Immunofluorescence staining was performed in the brain using the astrocyte marker GFAP, pericyte marker CD31, endothelial cell marker claudin 5, and extracellular matrix (ECM) markers laminin α4 and α5. Original magnification, 200×. Scale bar = 50 μm. (**F**) Representative TUNEL-stained brain section. Original magnification, 200×. Scale bar = 50 μm. (**G**) Representative Western blot of brain lysis: brain of ω-3 PUFA IR + CM shows decreased expression levels of Bax, cleaved caspase-3, and increased Bcl2 compared with the brain of WT IR + CM. Bar represents mean ± SD. WT, wild-type sham; ω-3, ω-3 PUFA oral-administration sham; WT IR + CM, IR renal injury in wild-type mice administered with CM; ω-3 PUFA IR + CM, IR renal injury in ω-3 PUFA oral-administration mice administered with CM. * *p* < 0.05, ** *p* < 0.01, *** *p* < 0.001.

ω-3 PUFAs increased the immunofluorescence intensity of the astrocyte marker GFAP, pericyte marker CD31, endothelial cell marker claudin 5, and ECM markers laminin α4 and α5 in the brains of ω-3 PUFA IR + CM mice compared with that in the brains of WT IR + CM mice (Figure 4A–E). The abundance of TUNEL-positive cells was increased in the brains of WT IR + CM mice compared with that in the brains of ω-3 PUFA IR + CM mice (Figure 4F). Furthermore, the protein-expression levels of Bax and cleaved caspase-3 were significantly decreased in the brain mice of ω-3 PUFA IR + CM mice as compared with those in the brains of WT IR + CM mice. However, Bcl2 was significantly increased in the brains of ω-3 PUFA IR + CM mice as compared with that in the brains of WT IR + CM mice (Figure 4G).

### 2.6. ω-3/Fat-1 Reduces JNK/p-38 Signaling in the BBB of Uremic Mice

**Figure 5:**
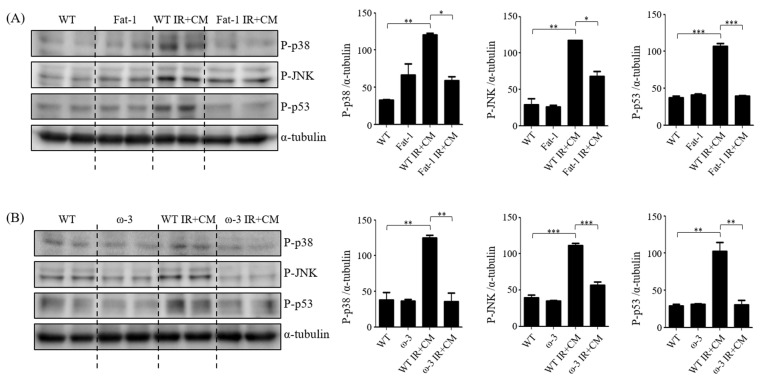
Effects of ω-3 PUFAs on JNK/p-38 signaling. (**A**) Representative Western blot of brain lysis: the brains of fat-1 IR + CM mice show decreased JNK, p38, and p53-expression levels compared with the brains of WT IR + CM mice. (**B**) Representative Western blot of brain lysis: the brains of ω-3 PUFA IR + CM mice show decreased JNK, p38, and p53-expression levels compared with the brains of WT IR + CM mice. Bar represents mean ± SD. WT, wild-type sham; fat-1, fat-1 induction sham; ω-3, ω-3 PUFA oral-administration sham; WT IR + CM, IR renal injury in wild-type mice administered with CM; fat-1 IR + CM, IR renal injury in fat-1 induction mice administered with CM; ω-3 PUFA IR + CM, IR renal injury in ω-3 PUFA oral-administration mice administered with CM. * *p* < 0.05, ** *p* < 0.01, *** *p* < 0.001.

ω-3 PUFAs, EPA and DHA exhibit various effects such as anti-inflammation and nerve protection against nerve cells. ω-3 PUFAs also reduce the expression of JNK/p38 MAPK and BBB permeability [22,23,24]. Therefore, the association of ω-3 PUFAs with JNK/p-38 signaling and neuroprotective effects in uremic and CM-induced BBB damage were investigated using brain tissues of ω-3 and fat-1 mice. The JNK/p38 MAPK family incorporates signals that affect cell proliferation, differentiation, survival, and migration. JNK activation increases phosphorylation and subsequent inactivation of p53, increasing resistance to apoptosis [25]. Protein expression of JNK, p38, and p53 in the brains of fat-1 IR + CM mice was significantly reduced compared with that in the brains of WT IR + CM mice (Figure 5A). The brains of ω-3 PUFA IR + CM mice had also significantly reduced expression of JNK, p38, and p53 compared with that of WT IR + CM mice (Figure 5B).

### 2.7. Survival Effect of ω-3 PUFAs in HBEC-5i Cells Treated with Indoxyl Sulfate (IS) and CM

**Figure 6:**
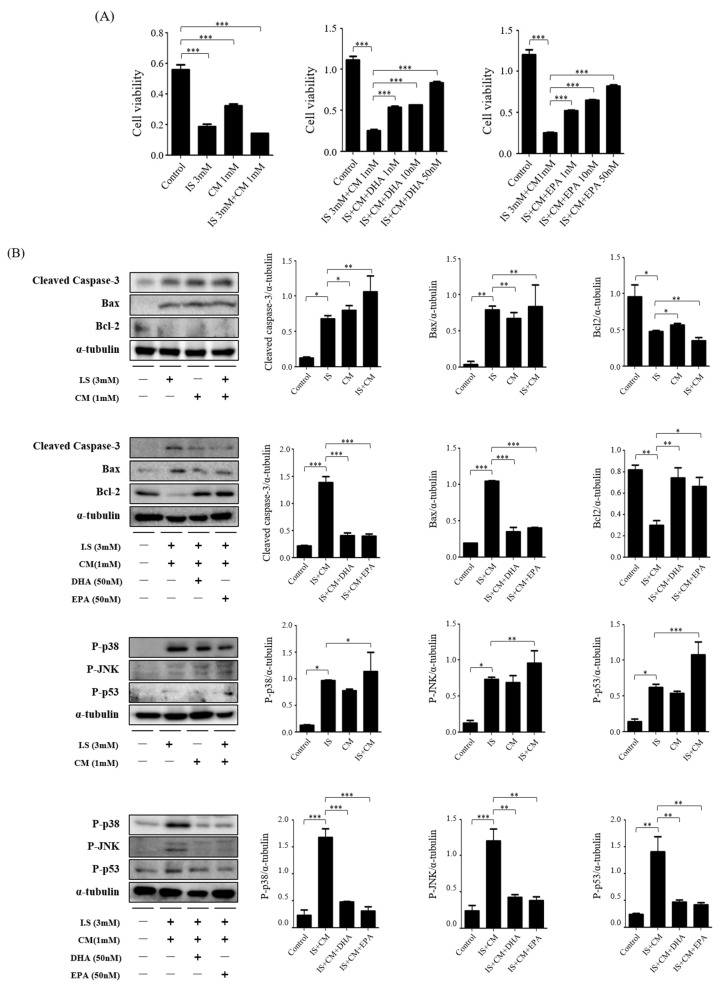
Effects of DHA and EPA on IS, CM, and JNK/p-38 activation toxicity in HBEC-5i. (**A**) IS and CM significantly reduced the survival of HBEC-5i cells in a dose-dependent manner. DHA and EPA increased the survival of HBEC-5i cells treated with IS and CM. (**B**) Representative Western blot: IS + CM-treated HBEC-5i cells had increased protein-expression levels of cleaved caspase-3, Bax, P-p38, P-JNK, and P-p53. DHA and EPA treatment reduces the protein expression of cleaved caspase-3, Bax, P-p38, P-JNK, and P-p53 in IS + CM-treated HBEC-5i cells. Bar represents mean ± SD. IS, indoxyl sulfate; CM, contrast medium (Omnihexol); DHA, docosahexaenoic acid; EPA, eicosapentaenoic acid. * *p* < 0.05, ** *p* < 0.01, *** *p* < 0.001.

HBEC-5i cells were exposed for 24 h to determine the toxicity of IS and CM. The protective effects of DHA and EPA on cell viability were determined using CCK-8 analysis. Treatment with IS and CM for 24 h showed a significant decrease compared with the control group. However, treatment with DHA and EPA for IS and CM treatment gradually increased (Figure 6A). HBEC-5i cells were seeded on the plate for 16–18 h to determine the effect of ω-3 PUFAs on toxicity in IS and CM. Then, they were exposed to IS, DHA, and EPA for 4 h and then treated with CM for 24 h. Compared with the control group, the IS + CM-treated group showed increased protein expression of cleaved caspase-3 and Bax but decreased protein expression of Bcl-2. When DHA and EPA were added to the IS + CM-treated group, protein expression of Bcl-2 significantly increased and protein expression of cleaved caspase-3 and Bax decreased. It was significantly increased compared with the IS + CM-treated group. Furthermore, to confirm that ω-3 PUFAs directly inhibit JNK/p38 signals, HBEC-5i cells were seeded on the plate for 16–18 h to determine the effect of ω-3 PUFAs on toxicity in IS and CM. Then, they were exposed to IS, DHA, and EPA for 4 h and then treated with CM for 2 h. Compared with the control group, the IS + CM-treated group showed increased protein-expression levels of P-p38, P-JNK, and P-p53. When DHA and EPA were added to the IS + CM-treated group, protein-expression levels of P-p38, P-JNK, and P-p53 significantly decreased (Figure 6B).

### 2.8. Survival Effect of ω-3 PUFAs in HT22 Cells Treated with IS and CM

**Figure 7:**
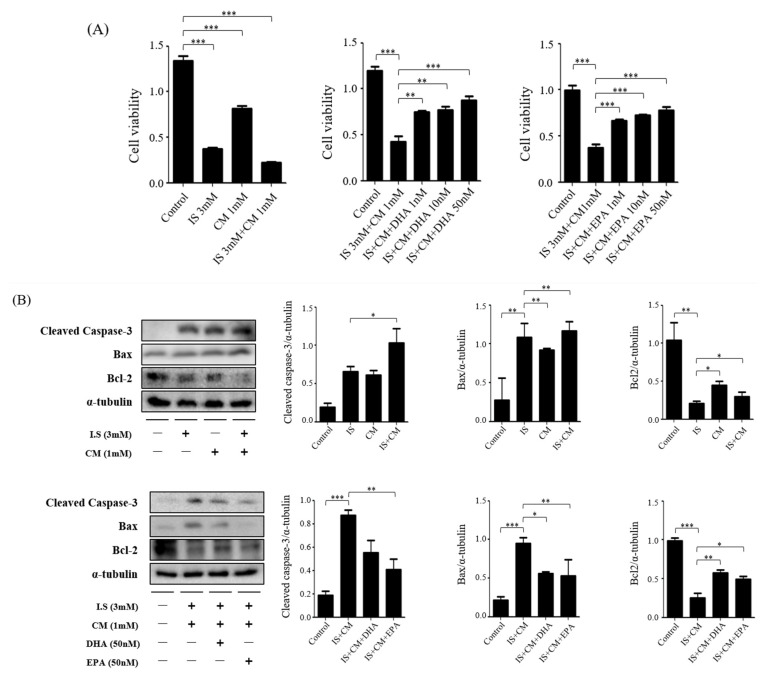
Effects of DHA and EPA on IS and CM in HT22. (**A**) IS and CM significantly reduced the survival of HT22 cells in a dose-dependent manner. DHA and EPA increased the survival of HT22 cells treated with IS and CM. (**B**) Representative Western blot: IS + CM-treated HT22 cells had increased protein-expression levels of cleaved caspase-3 and Bax but decreased protein-expression levels of Bcl-2. DHA and EPA treatment reduced the protein-expression levels of cleaved caspase-3 and Bax in IS + CM-treated HT22 cells and increased the protein-expression level of Bcl-2. Bar represents mean ± SD. IS, indoxyl; CM, contrast medium (Omnihexol); DHA, docosahexaenoic acid; EPA, eicosapentaenoic acid. * *p* < 0.05, ** *p* < 0.01, *** *p* < 0.001.

In HT22 cells, the toxicity of IS and CM and protective effects of DHA and EPA were identified in 24 h, and cell viability was determined using CCK-8 analysis. Treatment with IS and CM for 24 h showed a significant decrease compared with the control group, and treatment with DHA and EPA for IS and CM treatment showed a gradual increase (Figure 7A). Furthermore, HT22 cells were seeded on the plate for 16–18 h to determine the effect of ω-3 PUFAs on the toxicity of IS and CM. Then, they were exposed to IS, DHA, and EPA for 4 h and then treated with CM for 24 h. Compared with the control group, the IS + CM-treated group showed increased protein-expression levels of cleaved caspase-3 and Bax but decreased protein-expression levels of Bcl-2. When DHA and EPA were added to the IS + CM treatment group, the protein-expression level of Bcl-2 significantly increased, whereas those of cleaved caspase-3 and Bax decreased (Figure 7B).

## 3. Discussion

**Figure 8:**
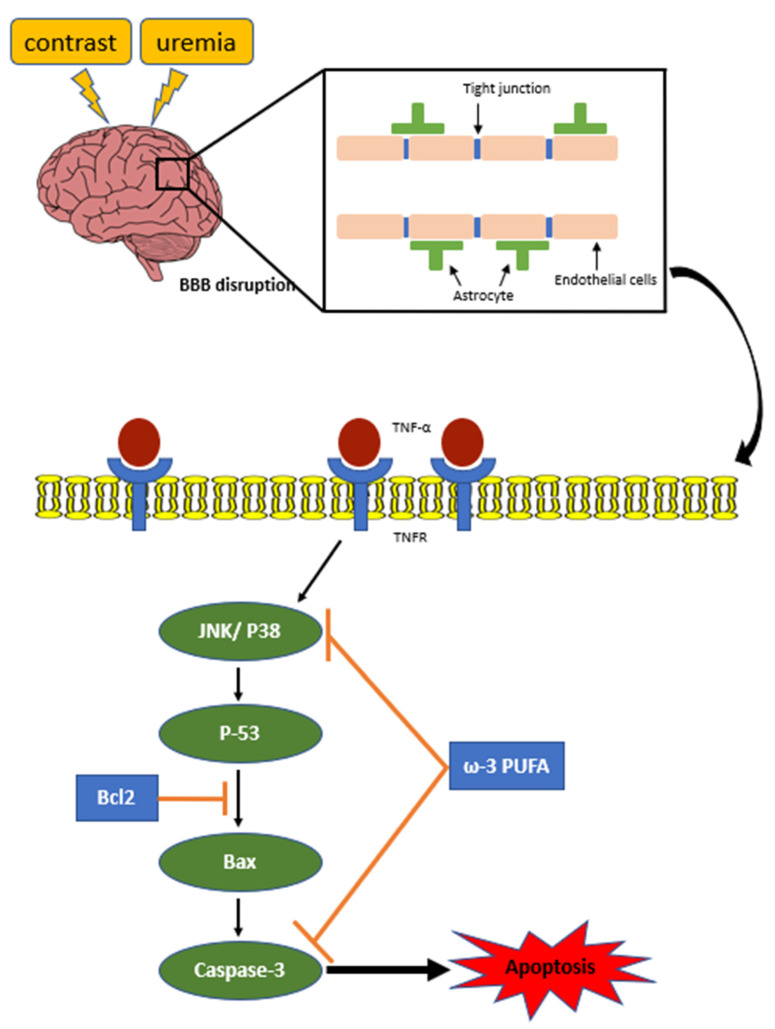
Schematic diagram. Ischemia–reperfusion (IR) increases kidney damage, and uremia induced by contrast medium (CM) causes BBB destruction and activates apoptosis. JNK/p38 signals involved in apoptosis activation cause neuroinflammatory and oxidative stress and result in BBB destruction. BBB damage caused by IR and CM in mice caused apoptosis and activated JNK/p38 signals. However, ω-3-PUFAs protected the BBB from IR and CM by inhibiting apoptosis and JNK/p38-signaling activation.

Here we demonstrated the CM induce BBB cell damage in uremic mice. And ω-3 PUFAs attenuates CM-induced BBB injury of uremic mice via JNK/p38-signaling suppression (Figure 8).

Generally, the BBB is assumed to be impermeable when administering CM iodide; however, damage to the BBB increases permeability, causing CM to penetrate the brain. In CKD, uremia-induced brain damage causes BBB destruction. Furthermore, CM administration was reported to increase brain nerve loss, apoptosis, and protein expression. However, ω-3 PUFAs are involved in BBB damage because this damage is weakened through JNK/p38-signaling suppression. These results can be used as a reference to prevent CKD-induced brain damage.

In addition, uremia has toxic effects on hippocampal cells [26,27,28], and it also leads to BBB damage including disruption of endothelial tight-junction proteins, inflammation, and vascular changes [7,8]. Furthermore, a study showed a higher risk of dementia after CM exposure, suggesting that CM will also affect the hippocampus area [29]. Although CM is neurotoxic, whether CM is toxic to the BBB is unclear. We investigated the staining intensity of Ki67 and NeuN in the brains of WT IR + CM mice, confirming that apoptosis of hippocampal neuronal cells and in the hippocampal region is weakened by CKD-induced uremia and CM administration. This shows an increase in hippocampal neuron cell damage and apoptosis when CM is administered to the CKD model.

The BBB makes brain capillaries impermeable when CM is administered [30]. Only cases where the BBB is severely damaged are detected, and most of them cannot pass if the BBB is not severely damaged [31]. We confirmed that the BBB cannot pass when CM is administered in the absence of brain damage. The BBB consists of major cell elements such as endothelial cells, astrocyte end foot, microglia cells, and pericytes [32]. By identifying a total of five BBB markers, GFAP (astrocyte cell marker), CD31 (endothelial cell marker and pericyte marker) [33], claudin-5 (endothelial cell marker and close connection protein) [34], laminins α4 and α5 (ECM protein) [35], we showed that they failed to pass when CM was administered in the absence of BBB damage.

As mentioned earlier, although iodized CM generally does not permeate the BBB, BBB damage can increase permeability, and CM exerts toxic effects on the brain [36]. The uremic toxin produced by CKD leads to BBB destruction and causes cognitive dysfunction and neurodegeneration [37]. Conversely, ω-3 PUFAs affect neurotransmitters and brain function, and ω-3 PUFAs intake increases learning, memory, and cognitive health. Animal studies have reported that a ω-3 PUFA-rich diet reduces the risk of BBB collapse [13,38]. Similarly, the results of this study suggest that ω-3 PUFAs improved the constituent cells of the BBB damaged by urea toxins. Increased antioxidant defense and reduced oxidative stress were reported in the ω-3 PUFA traumatic-brain-injury mouse brain [39], and apoptosis of weakened neuronal cells induced by uremia in ω-3 PUFA uremic mice and fat-1 uremic mouse brains [40]. In this study, we show that ω-3 PUFAs are involved in cell survival through decreased apoptosis marker expression in the uremic mouse brain. Through this, ω-3 PUFAs showed a protective effect on nerve cell damage and apoptosis induction in the BBB region caused by CKD and CM administration.

JNK/p38 signaling is an important stress-response kinase activated by various forms of damage, including ischemia. JNK signaling causes neuroinflammation, BBB degradation, and apoptosis on the postpartum day-two rat-baby model, and reduced JNK activation in the subarachnoid hemorrhage model alleviates BBB preservation and cerebral edema [15,41]. Furthermore, the p38 MAPK pathway can promote BBB destruction with secondary vascular edema in the transient focal cerebral ischemia (tFCI) rat brain cortex, and superoxide anions can stimulate p38 signals after IR damage [42]. This study shows that ω-3 PUFAs increased the survival rate and anti-apoptosis role in impaired BBB through the inhibition of JNK/p38 activation.

In a model using mouse cerebral endothelial cell lines (bEnd.3), a study reported that IS exhibited a direct toxic effect on hippocampal nerve cell lines, along with inducing oxidative stress via ROS production [27,43]. The iodized CM showed that exposure to iopamidol (300 mgI/mL) for 30 min in brain endothelial cells in mice could cause barrier dysfunction independent of osmotic pressure [44]. Cell death increased in HT22 (hippocampal cell lines) and HBEC-5i cells (BBB cell lines) treated with IS, CM alone, or both IS and CM. Considering brain results in uremic mice, DHA and EPA inhibited apoptosis and JNK/p38 signaling in the additional treatment groups. Thus, we report that ω-3 PUFAs act directly on neuronal cells in the BBB and hippocampal regions and show that ω-3 PUFAs have a protective effect against their toxicity.

This study showed that the BBB is destroyed by uremic toxin and CM, increasing apoptosis induction and neuroinflammation as CM passes through the destroyed BBB and reducing endothelial cell proliferation in the uremic mouse brain. In contrast, ω-3 PUFAs play a major protective role against BBB damage by exhibiting the attenuation effect of apoptosis and neuroinflammation induced by JNK/p38-signaling inhibition.

## 4. Materials and Methods

### 4.1. Cell Culture and Drug Treatment

Human cerebral microvascular endothelial cells (HBEC-5i cells) were incubated with Dulbecco’s modified Eagle’s medium F12 (DMEM/F12; WELGENE, Gyeongsan-si, Republic of Korea) comprising 10% fetal bovine serum (Life Technologies Inc., Gaithersburg, MD, USA) and 1% Anti-Anti at 37 °C under 5% CO2. HT22 cells, a mouse hippocampal neuronal cell line, were incubated with DMEM (WELGENE, Gyeongsan-si, Republic of Korea) comprising 10% fetal bovine serum (Life Technologies Inc., Gaithersburg, MD, USA) and 1% Anti-Anti at 37 °C under 5% CO2. HT22 and HBEC-5i cells were plated and made to adhere (16–18 h) and treated with IS (Alfa Aesar, Lancashire, England, UK) (3 mM) for 4 h and then treated with CM (Omnihexol 300, Korea United Pharm Inc., Seoul, Republic of Korea) (1 mM) for 2 or 24 h. To determine the effects of DHA and EPA (Sigma-Aldrich, St. Louis, MO, USA) on neurons and the BBB, cells were treated with IS for 4 h, followed by treatment with CM for 2 or 24 h. Cell viability was evaluated using a CCK-8 assay, according to the manufacturer’s protocol (Dojindo Molecular Technologies, Inc., Kumamoto, Japan). CCK-8 was added into each well and then incubated for 1 h at 37 °C before measurement. Absorbance at 450 nm was detected using a microplate reader (Multiskan™ FC; Thermo Fisher Scientific, Waltham, MA, USA). In addition, these cells were harvested and subjected to molecular analysis for p-JNK, p-38, and apoptosis.

### 4.2. Animal Model

**Figure 9:**
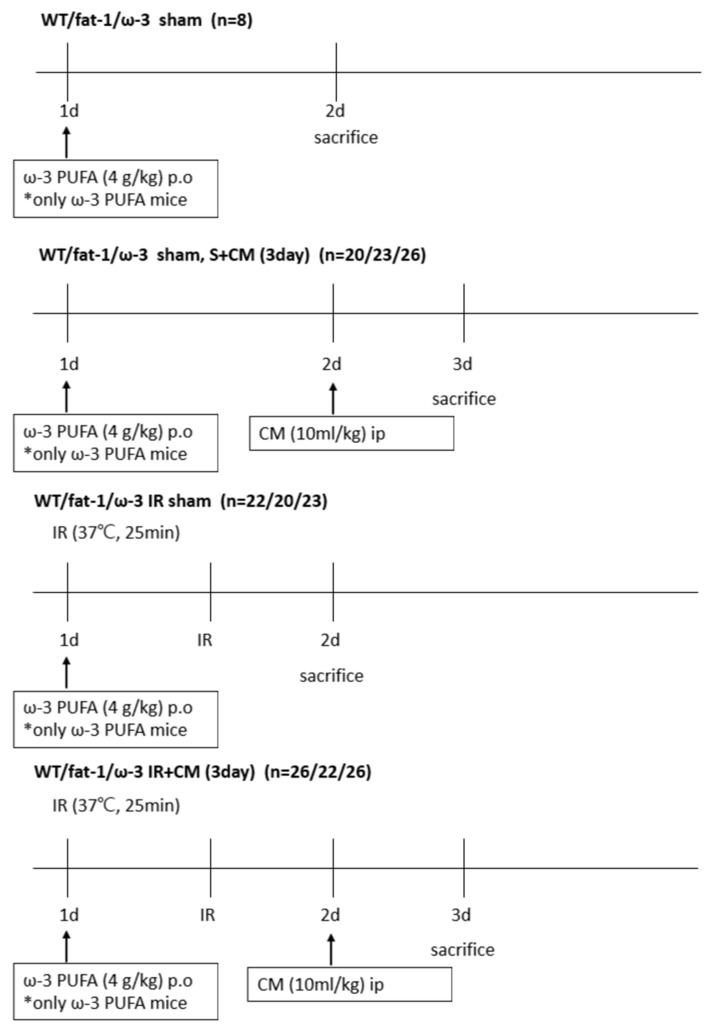
Illustration of the experimental schedule of mice. Schematic diagram of the experimental schedule. ω-3 PUFA mice receive 4 g/kg per os (PO) 24 h before all experimental schedules. The S + CM group received 10 mL/kg perfluorooctane sulfonate (ip) 24 h before the sacrifice, and the IR + CM group mice received 10 mL/kg perfluorooctane sulfonate (ip) 24 h after IR.

Male C57BL/6 mice (eight weeks old) were purchased from SAMTAKO Bio Korea (Gyounggido, Republic of Korea) and male fat-1 transgenic mice (b6 background, 8 weeks old) were provided by Dr. Jing Xuan Kang of Harvard Medical School (Boston, MA, USA). All transgenic fat-1 mice used were homozygous, and the absence or presence of the fat-1 gene in each mouse was assured by genotyping. In the ω-3 PUFA-treated groups, IR was induced after the oral administration of ω-3 PUFAs (4 g/kg) (United Pharmaceutical, Songpa-gu, Republic of Korea) to C57BL/6 mice 24 h ago. Food and water were freely consumed, and the mice were housed in a room maintained with a 12/12 h light/dark cycle. All animal experiments were conducted with the approval of the Animal Use and Care Committee at the Chungnam National University School of Medicine (202112A-CNU-203). The mice were divided into six groups: wild-type (WT) sham (n = 8), ω-3 PUFA sham (n = 8), fat-1 sham (n = 8), WT S + CM (n = 20), ω-3 PUFA S + CM (n = 26), fat-1 S + CM (n = 23), WT IR (n = 22), ω-3 PUFA IR (n = 23), fat-1 IR (n = 20), WT IR + M (n = 26), and ω-3 PUFA IR + CM (n = 26), and fat-1 IR + CM (n = 22). The mice were anesthetized with an intraperitoneal injection of ketamine (60 mg/kg). IR injury was performed as described previously [45]. After an abdominal incision, both renal pedicles were clamped bluntly. During the procedure, the clamps were removed 25 min after ischemia while the body temperature (37 °C) was maintained with the heat pad. Thereafter, all mice were sacrificed, and blood, kidney tissue, and brain tissue were collected 24 h after CM treatment for S + CM, 24 h after the procedure for IR, and CM treatment 24 h after the procedure for IR + CM (Figure 9).

### 4.3. Blood and Tissue Preparation

Tissues were prepared as described previously [46]. Blood was collected from the inferior vena cava of the anesthetized mice. The blood sample was placed in microcentrifuge tubes (4 °C). For BUN and serum creatinine (s-Cr), aliquots of serum were analyzed using a chemistry auto-analyzer, Toshiba 200FR (Toshiba Medical Systems Co., Tokyo, Japan). The kidney was fixed in 4% paraformaldehyde (4% PFA) at 4 °C and then embedded in Paraplast (Sherwood Medical, St. Louis, MO, USA) for light microscopy. The brain was perfused transcardially with 4% of PFA in PBS, and the tissues were fixed in 4% PFA for 16–18 h at 4 °C. The brain was removed, dehydrated, embedded with optimal cutting temperature (OCT) compound, frozen, and sectioned. The frozen section had a thickness of 10 μm.

### 4.4. Tissue-Injury Score

The kidney tissue was made into paraffin blocks, cut into 4 μm, and attached to a slide glass. The sections were deparaffinized with xylene, stained with H&E, and examined under a microscope (Olympus BX51, Olympus, Tokyo, Japan). Six consecutive fields were examined at 200× magnification and tissue-injury scores were averaged per slide. For the H&E sections, renal cortical vacuolization, proximal tubule simplification, renal cortical vacuolization, and peritubular/proximal tubule leukocyte infiltration were evaluated and scored as follows: normal = 0, <25% injury = 1, >25–50% injury = 2, and 50–75% injury = 3, >100% injury = 4. The injury scoring in H&E stain was evaluated by an experienced pathologist blindly; Masson’s trichrome staining was used to measure the inflammatory-cell accumulation and collagen deposition in the kidney-tissue sections [47].

### 4.5. Western Blot Analysis

The proteins were extracted with buffer containing 1 M PBS, 5 Mm EDTA, and 0.5% Triton X-100. After centrifugation (13,000 rpm for 10 min, 4 °C), the supernatant was collected for Western blot analyses. Protein (20 µg/lane) was electrophoresed on 10–15% SDS gel and then transferred to polyvinylidene fluoride membranes. The membranes were blocked with 5% non-fat dry milk for 1 h at room temperature (RT) and then incubated with primary antibodies against α-tubulin, Bcl2, Bax, cleaved caspase-3, p-p38, p-JNK, and p-p53 (1:1000, 1:1000, 1:1000, 1:1000, 1:1000, 1:1000, and 1:1000, respectively, Cell Signaling Technology, Danvers, MA, USA) at 4 °C overnight. The membranes were incubated with HRP-conjugated anti-rabbit IgG secondary antibodies (1:2000, Abfrontier Co., Ltd., Seoul, Republic of Korea) and HRP-conjugated anti-mouse IgG secondary antibodies (1:2000, Abfrontier Co., Ltd., Seoul, Republic of Korea) for 2 h at RT. The protein bands were visualized using a chemiluminescence detection kit (Thermo Scientific, South Logan, UT, USA). The same membranes were subsequently used for α-tubulin immune detection, and equal protein loading was ensured. The optical density for quantification was obtained using Gel-Pro Analyzer version 3.1 (Media Cybernetics, Silver Spring, MD, USA).

### 4.6. Terminal Deoxynucleotidyl Transferase dUTP Nick End Labeling Staining

Frozen sections (10 μm) were incubated in a 60 °C oven for 2 h and incubated in 4% PFA for 15 min at RT. Endogenous peroxidase was blocked with 3% hydrogen peroxide diluted in PBS. The sections were tested for TUNEL staining using an In Situ Cell Death Detection Kit (Fluorescein) (11684795910, Roche, Mannheim, Germany), following the manufacturer’s recommendations. The TUNEL-positive cells were identified with fluorescent signals using a fluorescent microscope. To evaluate apoptosis semi-quantitatively, six microscopic fields in the microscopic section were selected randomly at 200× magnification. The apoptosis index (number of TUNEL-positive cells/DAPI-positive cells) was calculated using Image Pro Plus 6.0 (Media Cybernetics, Silver Spring, MD, USA).

### 4.7. Immunofluorescence Staining

The section was incubated with primary antibodies against Ki67 (1:200, Abcam, Cambridge, UK), NeuN (1:100, ABN90, EMD Millipore, Burlington, MA, USA), GFAP, CD31, claudin 5, laminins α4, and α5 (1:200, Thermo Scientific, South Logan, UT, USA) at 4 °C overnight. It was further incubated with secondary antibodies against goat Alexa Fluor^®^ 488-conjugated anti-rabbit antibody(Thermo Scientific, South Logan, UT, USA), goat Alexa Fluor^®^ 594 conjugated anti-mouse antibody(Thermo Scientific), goat Alexa Fluor^®^ 594 conjugated anti-rat antibody(Thermo Scientific) prepared at 1:500 dilution in PBS containing 0.3% Triton X-100 at RT dark for 2 h, coverslipped with Fluoroshield™(Thermo Scientific) with 4′,6-diamidino-2-phenylindole (DAPI, H-1200-10, VECTASHIELD, Burlingame, CA, USA), and was then observed under a fluorescent microscope.

### 4.8. Statistical Analysis

All data were expressed as means ± standard deviations. Multiple comparisons among groups were analyzed using the one-way analysis of variance with a post-hoc Bonferroni correction. IBM SPSS Statistics for Windows version 20.0 (IBM Corp., Armonk, NY, USA) was used. Differences among the groups were considered significant at *p* < 0.05.

## 5. Conclusions

This study demonstrated that uremic toxin and CM damage the BBB, causing brain-cell death, and ω-3 PUFAs by reducing apoptosis attenuate this BBB damage. The protective mechanism of these effects on fat-1 mice and ω-3 PUFA mice was likely to be related to the JNK/p38-signal inhibition of apoptosis activation by increased P-JNK and P-p38. Thus, this study clearly indicated that ω-3 PUFAs play a protective role in CM-induced BBB damage in uremic mice.

## Data Availability

The datasets used and analyzed in the current study are available from the corresponding author upon reasonable request.
